# Pseudoaneurysm as a complication of ankle arthroscopy

**DOI:** 10.4103/0019-5413.58614

**Published:** 2010

**Authors:** Olubusola A Brimmo, Selene G Parekh

**Affiliations:** Department of Orthopaedic Surgery, St. John's Mercy Medical Center, St. Louis University, St. Louis, MO, USA; 1 Fuqua Business School, Duke University, Durham, NC, USA

**Keywords:** Ankle arthroscopy, anterior tibial artery, pseudoaneurysm

## Abstract

We present a case of a pseudoaneurysm of the anterior tibial artery following ankle arthroscopy with synovectomy, an extremely rare complication when standard anteromedial and anterolateral portals are used. The patient was diagnosed and treated with appropriate interventions which led to an uneventful recovery. Nevertheless, the potential sequelae of delayed diagnosis or misdiagnosis of the complication are dangerous; therefore, a high index of suspicion for a pseudoaneurysm must be maintained in the postoperative period.

## INTRODUCTION

Pseudoaneurysms are rare complications of foot and ankle arthroscopy, but may cause significant morbidity when they occur, with severe complications if left untreated. Ankle arthroscopy carries a lower risk of vascular complications when compared to the knee^1^ and is considered to be relatively safe from vascular injury, with the majority of complications being neurologic in origin.

There are six cases in the literature of anterior tibial artery pseudoaneurysms following ankle arthroscopy.^2^ This report describes a case of a pseudoaneurysm of the anterior tibial artery occurring after arthroscopy of the ankle, subsequent interventions, and current treatment options.

## CASE REPORT

**Figure 1:**
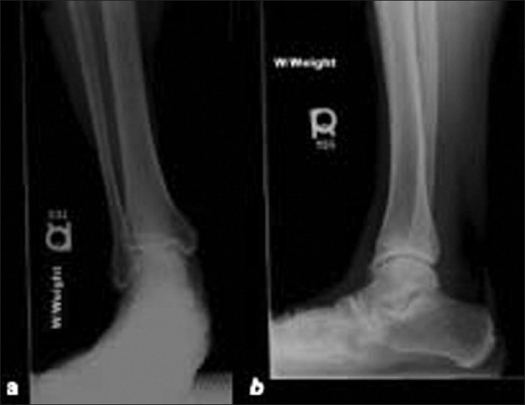
(a) Weight-bearing anteroposterior and (b) lateral radiograph of the right ankle demonstrating an anterior ankle osteophyte with medial talar dome sclerosis, suggestive of a medial talar dome osteochondral defect

**Figure 2:**
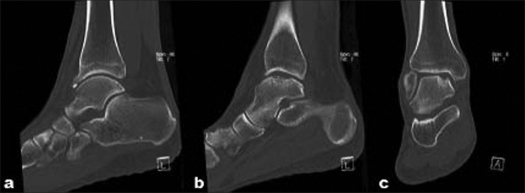
(a, b) Sagittal and (c) coronal CT scan of the right ankle showing osteophytes, a large medial talar dome osteochondral defect, and multiple loose bodies

A 36-year-old male presented with right anterior ankle pain which had been intermittently occurring for several years following a right ankle fracture. Physical examination revealed a pes planus deformity of the right ankle with tenderness over the anterolateral joint line, the peroneals, and the anterior talofibular ligament. Ankle movements were limited to 2° of dorsiflexion and 20° of plantarflexion. Radiographs of the right ankle demonstrated osteophyte formation in the anterior ankle with medial talar dome sclerosis, suggestive of a medial talar dome osteochondral defect [Figure 1a, b]. A CT scan of the right ankle showed a large medial talar dome osteochondral defect, multiple loose bodies, and osteophytes [Figure 2a–c]

The patient failed conservative therapy with a lace-up ankle brace and 2 months later underwent an arthroscopic right ankle synovectomy and a medial osteochondral defect microfracture through standard anteromedial and anterolateral portals. The lateral incision was extended to resect the anterior tibial osteophyte due to its large size. Ten days following his surgery, the sutures were removed and an elasticized cloth bandage was placed at the level of the ankle joint to hold dry dressings in place. The patient was instructed to remove the dressing after 24 h. The patient remained non-weight-bearing for the first 6 weeks following his ankle arthroscopy.

At his sixth week visit, the patient still had the previously placed elasticized cloth bandage and dressing in place. The patient had not removed this dressing since his second week visit. The ankle was extremely edematous at this time. The patient was instructed to bear weight as tolerated, start formal physical therapy, and participate in edema mobilization exercises. By week 11, most of the surrounding edema had resolved. However, on examination of the ankle, a pulsatile mass was appreciated on the anterior aspect of the ankle. The suspected differential diagnoses at this time were ankle joint effusion/infection, adhesions synovitis and a pseudoaneurysm. An MRI was ordered to investigate the etiology.

**Figure 3:**
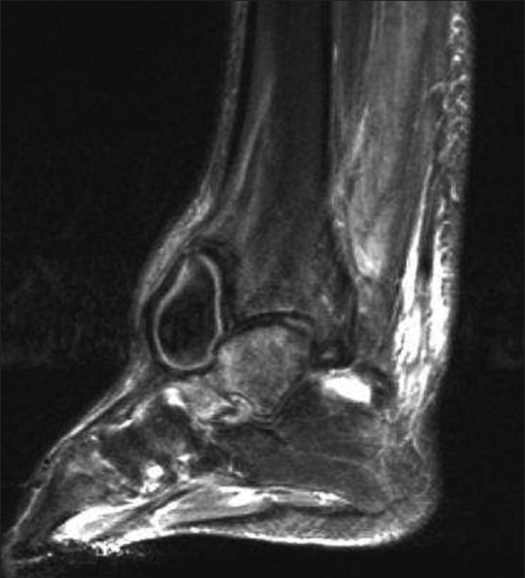
Sagittal MRI of the right ankle confirming the diagnosis of a pseudoaneurysm arising from the anterior tibial artery as it crossed the ankle joint

**Figure 4:**
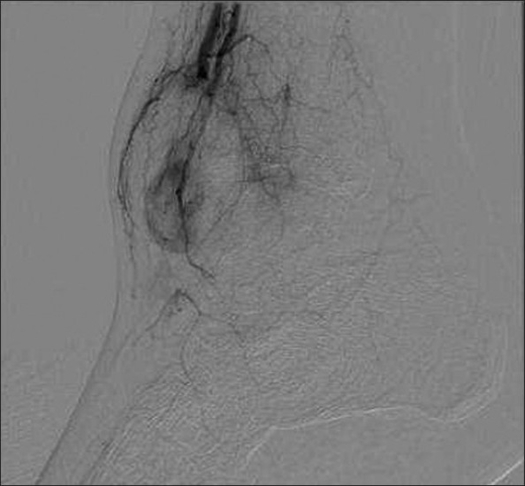
Arteriogram of the right lower extremity confirming the diagnosis of a pseudoaneurysm arising from the anterior tibial artery as it crossed the ankle joint

An MRI and duplex scan (Doppler ultrasonography) of the right ankle confirmed the diagnosis of a pseudoaneurysm arising from the anterior tibial artery as it crossed the ankle joint [Figure 3]. The patient was referred to the vascular surgery team, where he underwent an arteriogram of the right lower extremity [Figure 4] with successful ultrasound-guided thrombin injection of the pseudoaneurysm. After the procedure, Doppler signals within the anterior tibial artery and dorsalis pedis artery were within normal limits. The patient's subsequent recovery was uneventful, after a 9-month follow-up.

## DISCUSSION

Pseudoaneurysms are relatively uncommon complication of foot and ankle surgery. The most common site is the femoral artery after cardiac catheterization. The incidence of pseudoaneurysms after an arthroscopic surgery is 0.008%, mostly involving the popliteal artery after knee arthroscopy.^1^

A pseudoaneurysm, also known as a false aneurysm, is the result of trauma to all three layers of an artery resulting in extravasation of blood, and the formation of a fibrous tissue capsule containing blood flowing outside the injured vessel lumen. This fibrous capsule is void of the natural three-layer architecture of a true aneurysm and expand until confined by the limits of adjacent structures, and is therefore more likely to rupture than true aneurysms.^3^ Pseudoaneurysms are usually iatrogenic in origin but has been known to arise from trauma.^3^,^4^ Anatomically, the anterior tibial artery is in close proximity to the anterior ankle joint capsule at the level of the talar neck, and runs deep down the superior and inferior retinaculum. The artery may be damaged during portal insertion, removal, or instrumentation through the portal.^5^ Anterocentral portal placement has been implicated in a higher incidence of an anterior tibial artery injury; therefore, anteromedial and anterolateral portals are commonly employed.^1^

When a pseudoanuerysm occurs after foot and ankle surgery, there is usually no immediate sign of vascular injury. Patients usually present with pain and/or swelling out of proportion to the time after surgery, with the evolution of a pulsatile mass over days to weeks (possibly months to years), which may or may not be tender. The involved vessels usually depend on the surgical site, with the anterior tibial/dorsalis pedis and lateral plantar arteries most commonly affected and the posterior tibial artery less involved.^4^ No cases have been reported involving the medial plantar artery following foot and ankle surgery, likely because the abductor hallucis, flexor hallucis longus, and quadratus plantae muscles all lie superficial to it, making injury less probable.

Anatomic variations of the anterior tibial artery were described by Huber,^6^ who noted a 5.5% lateral deviation and 3.5% medial deviation. Mariani *et al*. concluded that lateral arterial position and aggressive synovectomy contributed to a case of anterior tibial artery pseudoaneurysm.^1^ Anterior tibial artery pseudoaneurysms have also been noted after ankle arthroscopy in a patient with hemophilia^7^ and a patient on anticoagulation,^2^ which were both considered to be partially responsible in the pathogenesis in each respective case. In this case, there were no arterial anatomic abnormalities, which would have been evident on the arteriogram and there were also no coexisting morbidities. It is possible that the anterior tibial artery was possibly injured during synovectomy, instrumentation through the portals.

Uncommon complications that have been described in the literature after pseudoaneurysms in the foot and ankle include hemarthrosis of the ankle^8^ and compartment syndrome.^9^ The most severe complication of an untreated psuedoaneurysm is a rupturing of this vascular structure. This can be problematic, leading to hemorrhaging into the soft tissue and hemodynamic instability in severe cases.^10^ Therefore, prompt diagnosis and timely treatment are in the patient's best interest.

As described in this case, a duplex ultrasound or angiogram will confirm the diagnosis of a pseudoaneurysm. Treatment depends on the particular vessel affected and may involve prolonged ultrasound-guided compression, thrombin injection, resection of the pseudoaneurysm with interpositional vein grafting or endovascular stenting, with thrombin injection the initial treatment of choice when possible, likely because it is less invasive than the other treatment options.^11^

## CONCLUSION

Pseudoaneurysms resulting from vascular damage are a rare but potentially dangerous complication of foot and ankle arthroscopy. Most cases will likely go undiagnosed initially due to their inherent slow progression. It is important to maintain a high degree of suspicion when a patient experiences abnormal pain, swelling, or recurrent hemarthrosis after ankle arthroscopy. When suspected, prompt diagnosis and treatment are warranted, with a referral to a vascular surgeon, when appropriate.
